# Preliminary In Vitro Wear Assessment of Ceramic Cemented Femoral Components Coupled with Polyethylene Menisci

**DOI:** 10.3390/ma14092112

**Published:** 2021-04-22

**Authors:** Saverio Affatato, Paolo Erani, Maurizio Fersini, Vincenzo Contaldi, Anna Rita Terrizzi, Antonio Licciulli

**Affiliations:** 1 Laboratorio di Tecnologia Medica, IRCCS Istituto Ortopedico Rizzoli, 40136 Bologna, Italy; paolo.erani@ior.it; 2 SALENTEC s.r.l., 73100 Lecce, Italy; maurizio.fersini@salentec.com (M.F.); vincenzo.contaldi@salentec.com (V.C.); 3 Dipartimento di Ingegneria Dell’Innovazione, Università del Salento, 73100 Lecce, Italy; annarita.terrizzi@unisalento.it (A.R.T.); antonio.licciulli@salentec.com (A.L.)

**Keywords:** knee arthroplasty, ceramic composite, X-ray diffractometry, SEM, roughness measurements, knee simulator, bovine calf serum, wear

## Abstract

Success of total knee replacement (TKR) depends on the prosthetic design and materials. The use of metal components is well established with the disadvantage of allergic reactions. Ceramics have been recently proposed because of high wear resistance, excellent biocompatibility, wettability, and suitable mechanical properties. This study was aimed at investigating in vitro wear resistance of Zirconia Toughened Alumina (ZTA)/Ultra-high-molecular-weight polyethylene (UHMWPE) of TKR femoral components. An in vitro protocol was designed with the application of relevant load profile, 6-degrees-of-freedom knee simulator, and 8 × 10^5^ cycles on the ZTA/UHMWPE configuration under bovine calf serum. Before and after wear test, the femoral components were investigated by using the Scanning Electron Microscope (SEM) and the X-Ray Diffraction (XRD) analyses, and stylus surface roughness measurements. The proposed pre-clinical test yielded repeatable results. In particular, gravimetric results showed that, after 8 × 10^5^ cycles, the mean weight loss of the polyethylene mobile components is 5.3 ± 1.1 mg. The surface roughness measurements (Ra_max_) performed after the wear test showed no significant variation on the UHMWPE menisci. A slight increase of roughness has been found on the ZTA (0.02 µm before wear test, 0.28 µm after the test). SEM observations did not show significant modification of the surface morphology. Tetragonal to monoclinic phase ratio was measured by XRD before and after wear test to evaluate stability of tetragonal ZrO_2_ phase. Minimal conversion of tetragonal to monoclinic phase was found from 5.4 to 8%. Although this study is a preliminary evaluation limited to in vitro tests, it provides novel pre-clinical indications about the potential of ceramic TKR femoral components.

## 1. Introduction

The knee joint is divided into two different sections: the patella and the tibial-femoral joint. The menisci are in-between them. The main movements of the knee are flexion and extension, a little degree of rotation is allowed also [1]. Knee osteoarthritis (OA), also known as degenerative joint disease, is typically the result of wear and tear and progressive loss of articular cartilage [2]. Typical knee symptoms such as pain and loss of mobility increase as the osteoarthritis progresses. When the symptoms can no longer be satisfactorily alleviated otherwise, the next step is a knee prosthesis implant.

Total knee replacement (TKR) is a common orthopedic surgery that involves replacing the femoral, the tibial component, and a tibial platform/insert/meniscus.

In other words, TKR is a surgical procedure in which an artificial joint or prosthesis replaces a damaged knee joint. The primary indication for TKR is pain, followed by functional limitation. Both femoral and tibial components are generally made of cobalt-chromium alloys (CoCr), whereas the tibial insert is made of ultra-high-molecular weight-polyethylene (UHMWPE). TKR is one of the most consolidated prosthetic surgeries to give pain relief and restoration of knee function. Many types of prostheses have been used for TKR during the last 40 years. Repeated cycles of failure and development led to fixed or mobile knee prostheses, total or unicondylar femoral components, cemented or cementless systems [3]. Yet this surgery remains one of the most expensive prosthetic surgeries, causing large costs to European countries [4].

Ceramic femoral components may be a viable alternative for all patients with allergies to metals, and may have better wear performance [5,6,7].

Femoral heads made of alumina ceramics were introduced in 1971 and femoral heads made of zirconia were introduced in 1985 for total hip arthroplasty [8,9]. Alumina ceramics are very interesting in clinical applications for their tribological properties due to their hardness. Some authors suggest that the use of zirconia may improve the mechanical strength of the ball head over alumina [10,11], while others state that zirconia may also reduce polyethylene wear with respect to alumina [12]. Mixed oxides ceramics have been indicated in the literature as a promising compromise between strength and wear requirements. Mixed-oxides ceramic materials can combine the properties of both alumina and zirconia. The addition of zirconia to alumina results in a composite material of increased toughness [8,13,14].

To the authors’ knowledge no investigations are available on mixed-oxides ceramics sliding against polyethylene menisci in TKR. Cristofolini and coworkers [15,16] tested the in vitro behavior of ceramic TKR femoral components. In particular, these authors compared the mechanical loosening between ceramic and metal femoral knee components.

The main aim of this work was to develop and validate a pre-clinical protocol to assess the wear behavior of new TKR components. In particular, ZTA knee femoral components were coupled with mobile UHMWPE menisci, and an in vitro protocol was designed to apply a relevant loading profile using a 6-degrees-of-freedom knee simulator for 8 × 10^5^ cycles. The samples were tested using a four-station displacement control knee joint simulator under bovine calf serum. In particular, the question was whether new ceramic knee femoral components would result in less wear than those usually made of metallic alloys. The study was also completed by performing the characterization of the new ceramic components at a microstructural scale (to correlate the composition and structure to the wear behavior) and at a macrostructural scale in order to assess surface morphology, homogeneity, roughness, and stability of such components under cyclic load.

## 2. Materials and Methods

A protocol was properly designed in order to test the ceramic knee femoral components under a cyclic loading onto a knee wear simulator.

### 2.1. Test Specimens

The wear behavior of new TKP mobile bearing configurations was investigated. In particular, three zirconia toughness alumina (ZTA 1, ZTA 2, ZTA 3) femoral components were coupled with three UHMWPE tibial inserts (1, 2, 3), and a fourth tibial insert was considered as insert soak control (4). The femoral components were CIM (Ceramic Injection Molding) manufactured by the company Salentec s.r.l. (Lecce, Italy); the material is based on Al_2_O_3_ (83%vol) and ZrO_2_ (17%vol) and the technological process has been developed in order to get high toughness, full density (up to 99.9% of theoretical density) sintered parts. The UHMWPE tibial inserts were designed by Salentec and manufactured by using the Computer Numerical Control (CNC DS Meccanica, mod. DS 150, Vicenza, Italy).

**Figure 1:**
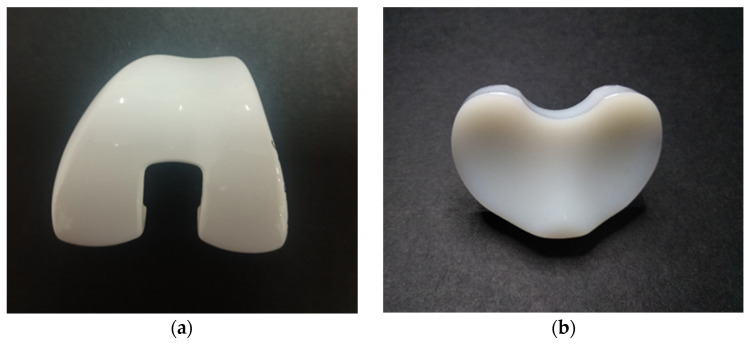
Zirconia Toughened Alumina (ZTA) femoral component (**a**) and UHMWPE tibial meniscus (**b**) before test.

A ZTA femoral component with a polyethylene meniscus before the wear test is shown in Figure 1.

### 2.2. Wear Tests

Wear tests were performed using a four-station knee simulator (Shore Western, Los Angeles, CA, USA) [17,18,19] as recommended by the International standard ISO 14243 (Implants for surgery–Wear of total knee-joint prostheses). The tibial trays and meniscal bearings were mounted in the lower tilting pools. The load was applied vertically to the tibial tray oscillating between 168 and 2600 N following a physiological profile. The applied kinematics was in displacement control [20] for the following degrees of freedom:

- flexion/extension angle oscillating between 0° (neutral) and 58° (flexion) synchronously with the load;
- anterior/posterior translation oscillating between 0.0 mm (neutral) and 5.2 mm (posterior);
- intra/extra rotation oscillating between 2.1° and 5.7°.

Four UHMWPE tibial components were soaked four weeks prior to the wear tests in order to achieve a steady level of fluid sorption, as recommended by the international standard; the liquid consisting of 25 per cent sterile bovine calf serum (Sigma, St. Louis, MO, USA) in deionized water; Ethylene-diamine-tetra-acetic acid (20 mmol/dm^3^) was added to minimize precipitation of calcium phosphates.

After this procedure, the prototypes were tested in the simulator (in dynamic mode) and the last one was used as a reference.

Each station was filled with lubricant (at 37 ± 2 °C), having the same composition as the one used during the soak.

The test duration was set at 8 × 10^5^ cycles. The frequency was set at 1.1 Hz in the range of ISO 14243-1 recommendations.

Weight loss on tibial menisci (1, 2, 3) was assessed at 2 × 10^5^ cycle intervals. At each stop the polyethylene tibial components were removed from simulator and cleaned using a detergent at 40 °C for 10 min. After ultrasonic cleaning in deionized water for 15 min, dried with a nitrogen flow, then dried in vacuum (pressure 0.1 bar) for 40 min. Each meniscus specimen was weighed three times using a semi-microbalance (Sartorius Cubis Mse 225 S-000-DU, Göettingen, Germany) with a sensitivity of 0.01 mg and an uncertainty of 0.01 mg. The wear trends determined from the weight loss of each polyethylene samples were corrected by the insert soak control (4). The ZTA femoral components (ZTA 1, ZTA 2, ZTA 3) were weighed before and at the end of the wear test, using an analytical scale (Gibertini E154, Modena, Italy) with a capacity of 150 g and resolution of 0.1 mg.

### 2.3. Characterization of Surface Roughness

Measurements of surface roughness were performed on the worn and unworn ceramic femoral components and tibial menisci in accordance with ISO 7207-2:2011(E) and Amd.1:2016(E), which specify the requirements for total and partial knee joint surfaces. A profiler (KLA-Tencor P-16+ / P-6 Profiler, Milpitas, CA, USA) was used.

Profiles were acquired and the maximum roughness (Ra_max_) was calculated as the average of the three measurements.

The Ra_max_ limits for ceramic and tibial components are respectively 0.1 µm (with cut-off of 0.25 mm) and 2 µm (with cut-off of 0.8 mm).

### 2.4. Surface Morphology Characterization

Microstructural analyses (surface morphology before and after the wear test) were performed on the bearing couple. In particular, the ceramic femoral components were examined by using scanning the SEM (JEOL JSM IT300LA, Tokyo, Japan); images were acquired by secondary and backscattered electron detectors (accelerating voltage of 10 kV, working distances of 11–13 mm).

Micrographs of UHMWPE tibial menisci were obtained with optical digital microscopy (Carl Zeiss Microscopy Axio Imager.A2m, Milano, Italy).

### 2.5. X-ray Diffractometry

The tetragonal to monoclinic conversion of zirconia after tribological test was evaluated by diffractometry (Rigaku Model D/MAX ULTIMA, Rigaku Corporation, Tokyo, Japan) comparing ZTA samples extracted from the femoral component before and after wear tests.

The scans were conducted using Cu-kα radiation, with a voltage of 40 kV and a current emission of 20 mA, in a range (θ–2θ) between 10 and 90° with a step size of 0.02° and acquisition of 2 s per step.

The monoclinic phase was quantified by the Toraya and Yoshimura method [21]. The fraction *X_m_* of monoclinic phase is given by Equation (1):

(1) $$X_{m}=\frac{I_{m}\left(\bar{1}11\right)+I_{m}\;\left(111\right)}{I_{m}\left(\bar{1}11\right)+I_{m}\;\left(111\right)+I_{t}\left(101\;\right)}$$

where *I_m_* $\left(\bar{1}11\right)$ monoclinic peak intensity (2θ ≈ 28.2°), *I_m_* (111) monoclinic peak intensity (2θ ≈ 31.3°), *I_t_* (101) tetragonal peak intensity (2θ ≈ 30.2°) [21,22]. The intensities were obtained after the baseline subtraction.

## 3. Results

### 3.1. Wear Quantitative Evaluation

All samples completed the wear test without failure. The weight values measured on femoral components before the test (0 cycles) and after the test (8 × 10^5^ cycles), are shown in Table 1.

Instead, each tibial component was weighed three times at 2 × 10^5^ cycle intervals, and the mean values of weight and weight loss measured are shown in Table 2.

It was observed that, at the end of the wear test, the weight loss measured on the ZTA knee components was 5 ± 2 mg (0.004% of initial weight), while the mean mass loss measured on the UHMWPE menisci was 5.3 ± 1.1 mg.

### 3.2. Surface Roughness

The roughness (Ra_max_) measured at 0 cycles is well below the limit imposed by the ISO 7207-2 standard requirements.

The roughness measurement (Ra_max_) was evaluated at 8 × 10^5^ cycles in the area where there was the highest contact pressure, and results are shown in Table 3.

The surface roughness on the ZTA parts at 8 × 10^5^ cycles exhibited a slight increase (from 0.02 µm before wear test to 0.28 µm after the test); no significant variation was observed on the UHMWPE meniscus.

### 3.3. Surface Morphology Characterization

**Figure 2:**
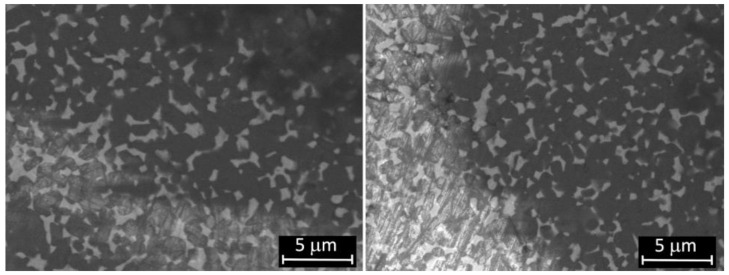
SEM micrographs of ZTA femoral surface before (**left**) and after (**right**) the test.

Micrographs of femoral condyle before and after wear test are shown in Figure 2. The surface morphology of the worn sample is almost similar to the unworn one, although there are some grooves due to the wear process, as also confirmed by the measured roughness after test. Some scratches present before and after wear tests have been ascribed to the action of polishing coarser abrasives granules during the manufacturing process.

**Figure 3:**
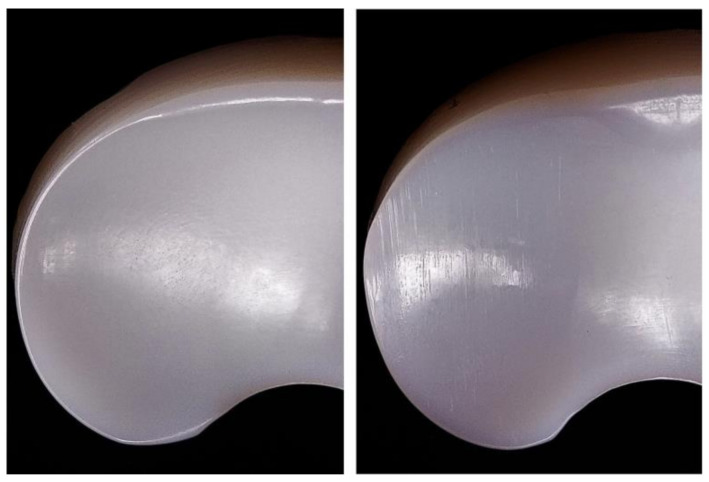
UHMWPE meniscus before (**left**) and after (**right**) the test.

**Figure 4:**
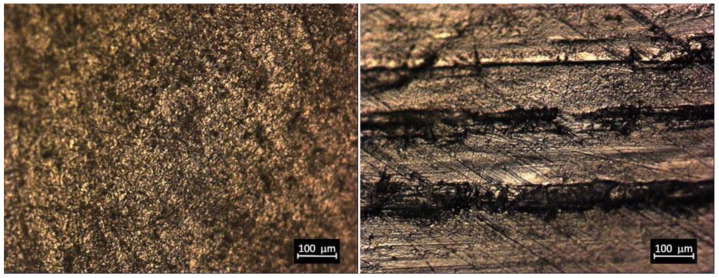
Micrographs of UHMWPE surface before (**left**) and after (**right**) the test (zoom 10X).

Polyethylene menisci were examined by visual and microscopic inspection. Figure 3 and Figure 4 show UHMWPE meniscus before and after test where scratches are evident on the surface of the sample after wearing cycles. Since the inspected ZTA femoral part is not altered by wear, it is unlikely that ceramic debris are formed during the test. Scratches are therefore attributed to the friction between ceramic and polyethylene.

### 3.4. X-ray Diffractometry Results 

Tetragonal phase destabilization on the surface of zirconia and ZTA with consequent formation of monoclinic phase is at the origin of the embrittlement of the components [23]. Tetragonal-to-monoclinic polymorphic transformation in ZTA femoral parts have been evaluated from XRD spectra.

The amount of monoclinic zirconia was quantified on the three samples before and after wear test to evaluate the stability of the tetragonal phase for ZTA under the test conditions. In Table 4 the percentage of monoclinic phase ranges from 5.4 (before test) to 8% (after test).

**Figure 5:**
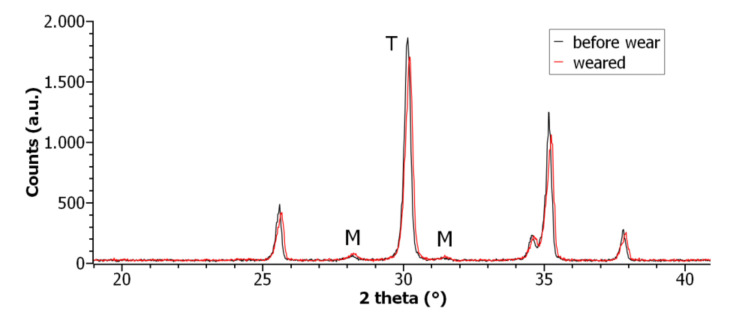
X-ray diffractograms obtained for ZTA femoral component.

The XRD spectra are shown in Figure 5. A slight increase of intensity is evident in the monoclinic peaks (2θ ≈ 28.2°and 2θ ≈ 31.3°) in the worn ZTA components, vice versa, a decrease in the intensity of the tetragonal phase peak (2θ ≈ 30.2°) is measured (with respect to the components before the wear test).

## 4. Discussion 

Wear behavior of ZTA knee femoral components coupled with mobile UHMWPE menisci is the focus of this study. The samples were tested using a knee joint simulator under bovine calf serum and cyclic load, to establish if ceramic femoral components could exhibit a lower wear than those made of metal alloys usually used for TKR.

Ceramic femoral components did not exhibit relevant wear as proven by absence of weight loss and SEM analyses. X-Ray diffractometry have relieved a minimal conversion of tetragonal to monoclinic zirconia phase: diffractograms show a decrease in the intensity of the tetragonal phase peak and a slight increase of intensity in the monoclinic peaks in the worn ZTA components with respect to the components before the wear test. The total phase change is not relevant to compromise characteristics and mechanical properties (mechanical strength and toughness) of the prostheses.

A real comparison is not possible because there are very few studies referred to ZTA ceramic knee components coupled with UHMWPE menisci. Zietz et al. [6] tested ceramic materials on knee simulator following the standard ISO 14243 and non-significant variation of roughness was measured on UHMWPE meniscus surface, as results found in this study. Cristofolini and coworkers [15], in order to explore the idea of introducing a ceramic TKR femoral component and prevent the risks of a possible clinical failure, developed an experimental protocol to evaluate implant–bone fixation.

The investigations performed in this study showed that the wear rate of the UHMWPE insert coupled with ZTA femoral components is less than metallic femoral components previously tested on the same knee wear simulator. Comparing the obtained results of this work with state of art in metallic knee femoral components coupled with UHMWPE, Affatato et al. [17] tested metallic knee femoral components against UHMWPE obtained a mean mass loss of 20.1 mg. The same author, in another work [24], investigated the wear behavior of mobile and fixed bearings, tested for 5 million cycles using bovine calf serum as lubricant and using the same simulator. It was found that mobile designs components showed lower weight losses than the fixed components but greater if compared with current results of this study. In a previous work, Jaber et al. [25] compared the wear behavior of total knee metallic femoral components coupled with UHMWPE menisci, simulating highly demanding daily load waveform, and found a mass loss, after 0.8 million cycles, greater of about 50% of the results obtained in the this work. In addition, Affatato and coworkers [26] tested small and large sized-mobile inserts (samples #2 and #6 respectively) on the same knee wear simulator for 2 million cycles, using bovine calf serum as lubricant. The results emphasized that the larger size (sample #6) had almost twice the weight loss with respect to the smaller one. Although consistent literature on TKR wear tests became available in recent years, to the authors’ knowledge, there are not many reports describing in detail both the wear performances of implants with different designs, tested with the same knee simulator.

Therefore, it is possible to emphasize from Table 2 that the gravimetric mass loss obtained in this study on the UHMWPE menisci is lower than the results of the previous discussed wear tests with metallic femoral component against UHMWPE menisci under bovine calf serum as lubricant and using the same knee simulator.

The present study obviously has some limitations. The first is that results found are based on a small number of specimens. The second is the fact that the ceramic knee components were compared only with metallic commercial knee prostheses. However, it represents a preliminary approach to understand wear behavior. Further clinical investigations are required to establish whether these differences in wear would result in different long-term outcomes.

## 5. Conclusions

Femoral knee implant plays a key role on ultra-high molecular weight polyethylene (UHMWPE) wear rates. In this work, ZTA femoral components wear and tribological properties have been investigated.

ZTA/UHMWPE were tested using a four-station knee simulator. The set up was used to simulate a 0.8 million walking cycles and provide a comprehension of TKR kinematics over time and relevant loading conditions. The test and investigations showed that the wear rate of the UHMWPE insert coupled with ZTA femoral components is extremely low compared to similar studies on metal femoral components. Not only the wear rate but also the modification of ZTA surface microstructure is negligible, as revealed by SEM and XRD.

This study underlines the excellent wear performances of ZTA/ UHMWPE TKR and opens up new perspectives on the use of ceramic materials for knee replacements.

Future investigations will be focused to analyze if the fixed or mobile knee prosthesis could have significant effects on the wear of UHMWPE inserts.

## Figures and Tables

**Table 1:** Weight and weight loss (± instrumental error) of femoral components.

ZTA	Weight (mg)		Weight Loss (mg)
Knee Femoral Components	0 Cycles	8 × 10^5^ Cycles	
ZTA #1	126,837 ± 1	126,831 ± 1	6 ± 2
ZTA #2	128,201 ± 1	128,192 ± 1	9 ± 2
ZTA #3	126,428 ± 1	126,418 ± 1	10 ± 2

**Table 2:** Mean weight and weight loss (± standard deviation) of tibial menisci.

UHMWPE Tibial Menisci	Weight (mg)				Weight Loss (mg)
	0 Cycles	2 × 10^5^ Cycles	4 × 10^5^ Cycles	8 × 10^5^ Cycles	
#1	23,493.3 ± 0.2	23,484.1 ± 0.4	23,478.3 ± 0.03	23,476.7 ± 0.3	7.7 ± 1.3
#2	23,700.1 ± 0.5	23,695.6 ± 0.4	23,692.7 ± 0.1	23,689.9 ± 0.2	1.4 ± 1.2
#3	23,508.9 ± 1.8	23,501.3 ± 0.1	23,496.7 ± 0.01	23,493.4 ± 0.5	6.7 ± 0.7

**Table 3:** Mean femoral and tibial specimens’ roughness (± standard deviation).

Specimens	Roughness (µm)	
	0 Cycles	8 × 10^5^ Cycles
ZTA Femoral Component	0.021 ± 0.004	0.3 ± 0.1
UHMWPE Tibial Meniscus	0.20 ± 0.04	0.17 ± 0.06

**Table 4:** Mean monoclinic zirconia content (± instrumental error) in ZTA femoral component.

Wear Test	% Monocline Phase
Before Wear Test	5.4 ± 0.3
After Wear Test	8.0 ± 0.4

## Data Availability

Data is contained within the article.
